# Structural Diversity of Class 1 Integrons and Their Associated Gene Cassettes in *Klebsiella pneumoniae* Isolates from a Hospital in China

**DOI:** 10.1371/journal.pone.0075805

**Published:** 2013-09-30

**Authors:** Bin Li, Yongfei Hu, Qi Wang, Yong Yi, Patrick C. Y. Woo, Hua Jing, Baoli Zhu, Cui Hua Liu

**Affiliations:** 1 CAS Key Laboratory of Pathogenic Microbiology and Immunology, Institute of Microbiology, Chinese Academy of Sciences, Beijing, China; 2 The 306 Hospital, Beijing, China; 3 State Key Laboratory of Emerging Infectious Diseases, Department of Microbiology, The University of Hong Kong, Hong Kong, Special Administrative Region, China; Robert Koch Institut, Germany

## Abstract

**Background:**

*Klebsiella pneumoniae* strains carrying class 1 integrons are becoming more common worldwide, and their role in the dissemination of drug resistance is significant. The aim of this study was to characterize the structural diversity of class 1 integrons and their associated gene cassettes in *K. pneumoniae* isolates from hospital settings.

**Methodology/Principal Findings:**

We analyzed a total of 176 *K. pneumoniae* isolates in a tertiary-care hospital in Beijing, China for the period of November 1, 2010-October 31, 2011. The presence of class 1 integrons and gene cassettes was analyzed by PCR and sequencing. The prevalence of class 1 integrons was 51.1% (90/176). Fourteen different gene cassettes and 10 different gene cassette arrays were detected. *dfrA* and *aadA* cassettes were predominant and cassette combination *dfrA1*-*orfC* was most frequently found (13.6%, 24/176). Strong association between resistance to a variety of drugs (both phenotypes and the associated genes) and the presence of class 1 integrons was observed. In addition, we also identified an association between some previously identified prevalent sequence types (such as ST11, ST15, ST147, ST562, and ST716) and the presence of class 1 integrons.

**Conclusions/Significance:**

Data from this study demonstrated that class 1 integrons are highly diverse and are associated with a variety of drug resistance phenotypes, drug resistance genes, as well as genotypes among *K. pneumoniae* isolates. Continuous monitoring of gene cassettes in class 1 integrons is warranted to improve the understanding and control of drug resistance among hospital settings.

## Introduction

The widespread use of antibiotics coupled with the intra- and inter-species transfer of resistance determinants mediated by plasmids, transposons and gene cassettes in integrons have contributed to the rapid transmission of drug resistance in bacterial pathogens, especially among members of the *Enterobacteriaceae* family [1-4]. The genetic organization of integrons can be quite diverse with different combinations of gene cassettes. There are 5 different classes of integrons, each encoding a distinct integrase gene. Class 1 integrons are the most common integron type present in clinical isolates of the *Enterobacteriaceae*. The most frequently identified gene cassettes within class 1 integrons in *Enterobacteriaceae* are those encode resistance to streptomycin (*aadA*) and trimethoprim (*dfrA*) [5]. More than 130 different gene cassettes containing various resistance genes have been identified, conferring resistance to different families of antibiotics such as aminoglycosides, β-lactams, chloramphenicol, trimethoprim, erythromycin, and rifampicin [6]. Moreover, detailed information of more than 8,550 gene cassettes (up to 16 May, 2013) have been collected in a web-based database INTEGRALL, which was created to congregate information on the phylogeny of the bacterial hosts and their ecology, the molecular diversity of inserted gene cassettes and the types of integrase (http://integrall.bio.ua.pt/) [7].

*K. pneumoniae* is an important opportunistic pathogen that causes urinary tract and intra-abdominal infections, neonatal meningitis and pneumonia in immunocompromised patients. *K. pneumoniae* strains carrying complex class 1 integrons are becoming more common. The recent emergence of multidrug-resistant (MDR), extensively drug-resistant (XDR), and pandrug-resistant (PDR) *K. pneumoniae* isolates has become a serious problem in healthcare settings worldwide including China. A strong relationship between MDR *Enterobacteriaceae* strains and the presence of integrons has been proven [8]. Since class 1 integron has been identified as a primary source of antimicrobial resistance genes and suspected to serve as reservoirs and exchanging platforms of resistance genes in a variety of Gram-negative bacteria, knowledge of the epidemiology and molecular characteristics of class 1 integrons in *K. pneumoniae* is essential for implementing intervention strategies. Though there have been a number of reports on class 1 integrons among different *Enterobacteriaceae* [9-11], little is known about the structural diversity of class 1 integrons and their associated gene cassettes in *K. pneumoniae* isolates from hospital settings in China. In our previous study, we detected a high percentage of class 1 integrons from clinical *K. pneumoniae* isolates and our MLST genotyping analysis data indicated that certain drug-resistant *K. pneumoniae* clones are highly prevalent [12,13]. We thus conducted this study to perform more in-depth analysis of the genetic structural characteristics of class 1 integrons and their associated gene cassettes among 176 *K. pneumoniae* isolates from the 306 Hospital, a tertiary care hospital in Beijing, China for the period of November 1, 2010 to October 31, 2011, to better understand the evolution and dissemination of drug resistance genes in *K. pneumoniae*.

## Methods

### Ethics statement

The investigation protocols used in this study were approved by the institutional ethics committee of the 306 Hospital, Beijing, China. Written informed consent for *K. pneumoniae* isolates to be collected as well as for their information to be stored in the hospital database for research purposes was provided by participants. Written informed consent was obtained from the next of kin, caretakers, or guardians on the behalf of the minors/children participants involved in this study. Permission for using the information in the medical records of the patients for research purposes was obtained from the 306 Hospital. The Institute ethics committee of the 306 Hospital reviewed that relevant ethical issues in this study were considered.

### Bacterial isolates

Since this is a further in-depth analyses of the genetic structural characteristics of class 1 integrons among *K. pneumoniae* isolates based on previous studies on the analysis of drug resistance determinants and MLST genotyping for *K. pneumoniae*, the isolates used in this study largely overlapped with those two previous studies [12,13]. This study included a total of 176 *K. pneumoniae* isolates collected from non-repetitive patients being treated in the 306 Hospital in Beijing, China for the period of November 1, 2010-October 31, 2011. Drug susceptibility testing (DST) for the *K. pneumoniae* strains was performed using the bioMérieux VITEK2 system following manufacturer’s instructions as described previously [12]. The ESBL production was tested by the bioMérieux VITEK-2 AST-GN13 test and the carbapenemase production was further confirmed for those carbapenemase gene positive strains by the double disk diffusion method [14]. Clinical records of patients were reviewed retrospectively. Drug resistance genes and class 1 integrons were detected by PCR and sequencing using 37 pairs of primers as described previously [12,15-37]. The detected resistance genes include: beta-lactamase genes (*bla*_TEM-like_, *bla*_SHV-like,_ *bla*_CTX-M-1-2-8-9-25-group_, *bla*_CTX-M-9_, *bla*_KPC-like_, *bla*_NDM-like_, *bla*_OXA-48-like_, *bla*_VIM-like_, *bla*_IMP-like_, *bla*_CMY-like_, *bla*_FOX-like_, *bla*_DHA-like_), PMQR genes (*qnrA/B/C/D/S*-like, *aac(6*')*1b-cr*, *qepA*), aminoglycoside resistance genes (*armA, rmtB, aacC1, aacC2, aacA4, aadB, aphA6*), and trimethoprim resistance gene (*dhfr*).

### Sequencing and bioinformatic analysis of class 1 integrons and gene cassettes

The gene cassettes of the class 1 integrons were amplified using specific primers for the 5´ and 3´ conserved segments (5´ CS and 3´ CS) as described previously [38]. The PCR products were sequenced and the complete sequence for each integron was obtained by using a primer walking method as described above. Resulting sequences were assembled by using SeqMan program within the Lasergene suite version 7 (DNAstar Inc, Madison, WI, USA). Potential open reading frames (ORFs) were predicted by using the NCBI (National Center for Biotechnology Information) ORF Finder tool (http://www.ncbi.nlm.nih.gov/gorf/gorf.html). BLAST (http://blast.ncbi.nlm.nih.gov/Blast.cgi) against GenBank database and The Integron Database INTEGRALL (http://integrall.bio.ua.pt/) were performed repeatedly for sequence comparison and annotation [39]. The nucleotide sequences of the integrons were deposited in the GenBank nucleotide sequence database and the accession numbers were obtained for each integron cassette array. In order to confirm whether some genes such as *aac(6*')*-Ib-cr* and *aacA4* were located within class 1 integrons lacking the 3´ CS, we used the 5’ CS primer coupled to a primer specific for the *aac*(*6*')*-Ib-cr* gene (CCATATGGGGTGGTTACGGT) or *aacA4* gene (CCATGTACACGGCTGGACC) for PCR and sequencing.

### Definitions

MDR was defined as acquired non-susceptibility to at least one agent in three or more antimicrobial categories, XDR was defined as non-susceptibility to at least one agent in all but two or fewer antimicrobial categories (i.e. bacterial isolates remain susceptible to only one or two categories) and PDR was defined as non-susceptibility to all agents in all antimicrobial categories [40]. The antimicrobial groups tested in this study include: cephalosporins, aminoglycosides, aminocyclitol antibiotic, carbapenems, fluoroquinolones, folate pathway inhibitors, cephamycins, nitrofuran antibiotics, penicillins, antipseudomonal penicillins+beta-lactamase inhibitors, penicillins+beta-lactamase inhibitors, ansamycins, monobactams. The antibiotics tested include: cefazolin, ceftriaxone, ceftazidime, cefepime, gentamicin, tobramycin, amikacin, streptomycin, spectinomycin, imipenem, ertapenem, ciprofloxacin, levofloxacin, trimethoprim cefotetan, nitrofurantoin, ampicillin, piperacillin/tazobactam, ampicillin/sulbactam, rifampicin, aztreonam.

## Results

### Class 1 integron structures and resistance genes

**Figure 1:**
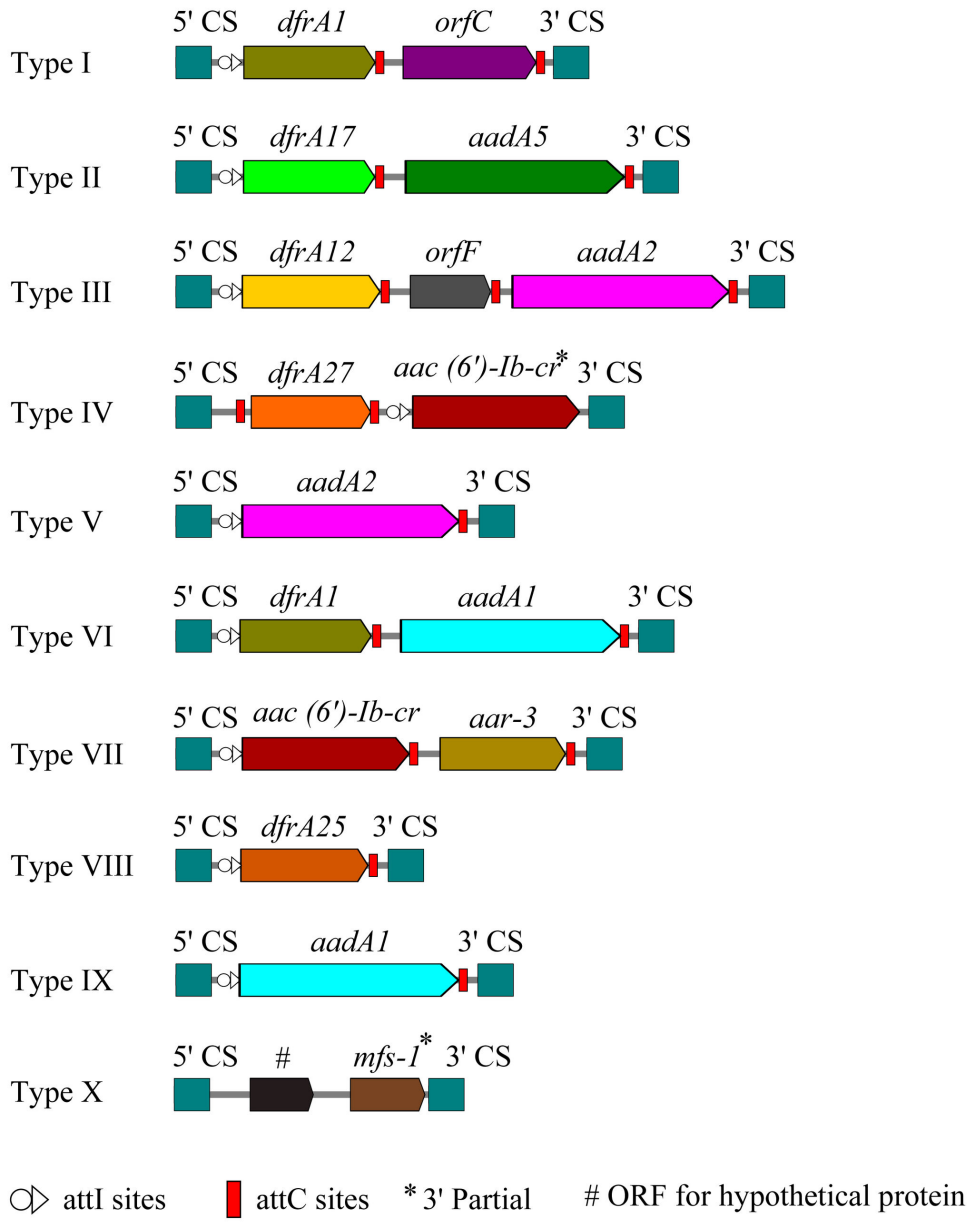
Schematic representation of the variable regions of class 1 integrons identified in *K. pneumoniae isolates*. Integron type with different cassette arrays (type I to X) are arranged as identified in Table 1. Gene cassettes are shown as boxes, with arrows indicating the orientation of transcription and hollow circles indicating the 59-base elements. The 5’- and 3’-conserved segments (5’-CS and 3’-CS) are annotated.

Ninety of the tested 176 isolates (51.1%) yielded a PCR product of the class 1 integron gene with variable lengths. Except for 20 isolates which yielded a 126-bp PCR product containing only the 5’-CS and the 3’-CS sequences of the class 1 integron, the remaining 70 isolates contained class 1 integrons with different gene cassette arrays. One isolate contained two class 1 integrons and all the other isolates contained only one class 1 integron each. Ten different class 1 integron gene cassette arrays, for which we classified as type I–X, were identified in the class1 integron positive isolates. They include: *dfrA1-orfC, dfrA1-aadA5, dfrA12-orfF-aadA2, dfrA27-aac(6*’)*-Ib-cr, aadA2, dfrA1-aadA1, aac(6*’)*-Ib-cr-aar-3, dfrA25, aadA1*, ORF for hypothetical protein*-mfs-1*. No gene cassettes encoding ESBLs or proteins associated with resistance to carbapenems were identified. Table 1 shows an overview of various gene cassette arrays detected in the *K. pneumoniae* isolates. Table 1 and Figure 1 show an overview and schematic representation of various gene cassette arrays detected in the *K. pneumoniae* isolates.

**Table 1:** Characteristics of class 1 integrons and their associated gene cassette arrays from 70 *K. pneumoniae* isolates (one of the isolates has two intergrons).

**Integron type (No. of isolates)**	**Gene cassette arrays**	**Amplicon size (bp)**	**Associated resistance phenotype**	**Accession No.**
I (24)	*dfrA1-orfC*	1171	Trimethoprim	JQ823008
II (12)	*dfrA17-aadA5*	1602	Trimethoprim, streptomycin, spectinomycin	JQ823009
III (10)	*dfrA12-orfF-aadA2*	1851	Trimethoprim, streptomycin, spectinomycin	JQ823010
IV (10)	*dfrA27-aac(6’)-Ib-cr ^a^*	1391	Trimethoprim, Fluoroquinolone	JQ823011
V (4)	*aadA2*	1011	Streptomycin, spectinomycin	JQ823012
VI (4)	*dfrA1-aadA1*	1519	Trimethoprim, streptomycin, spectinomycin	JQ823013
VII (3)	*aac(6’)-Ib-cr-aar-3*	1466	Fluoroquinolone, rifampicin	JQ823014
VIII (2)	*dfrA25*	719	Trimethoprim	JQ823015
IX (1)	*aadA1*	966	Streptomycin, spectinomycin	JQ823016
X (1)	ORF for hypothetical protein, *mfs-1* ^a^	812	Unknown	JQ823017

a partial gene.

Table S1 shows the resistance genes that were not located within class 1 integrons including ESBL genes, PMQR genes, as well as those encoding resistance to carbapenems, aminoglycosides, and folate pathway inhibitors. The proportion of a variety of drug resistance genes were significantly higher among class 1 integron positive isolates as compared to class 1 integron negative isolates, and they include the following ones: *bla*_CTX-M-1,_ *bla*_CTX-M-3,_ *bla*_CTX-M-8,_ *bla*_CTX-M-10,_ *bla*_CTX-M-15_ *, bla*_OXA-48,_ *dhfr, qnrB, qnrD, qnrS, aac(6*’)*-Ib-cr, aacA4, aacC2, aadA1.*

**Figure 2:**
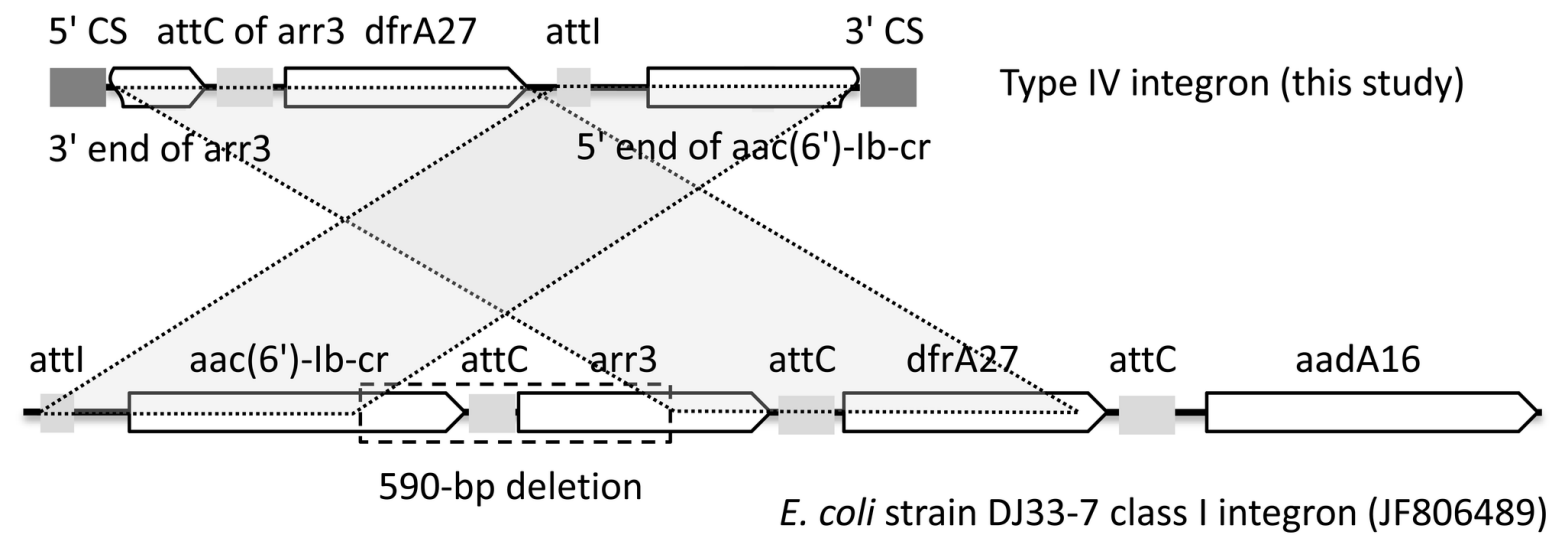
Comparison of the type IV integron in this study with its *E. coli* homologue in database. Sequence of *E*. *coli* strain DJ33-7 class I integron (JF806489) was retrieved from NCBI. The type IV integron (JQ823011) is arranged as indicated in Table 1. Gene cassettes are shown as boxes, with arrows indicating the orientation of transcription. The 5’- and 3’-conserved segments (5’-CS and 3’-CS), attC, attI sites are annotated. Dashed box indicates a 590-bp deletion in the type IV integron.

To investigate the distribution of the detected class 1 integrons with prevalent arrays in the genomes among diverse bacterial species of phylogenetically distant bacteria including Gram-positive and Gram-negative bacteria, we conducted BLAST search against the available databases GenBank and INTEGRALL, using strict filter parameters of more than 99% nucleotide identity and at least 80% query coverage. The results indicate that, most types of the class 1 integrons are presented in diverse bacterial species. For example, the type V integron carrying gene *aadA2* was detected in 29 different species, the type III carrying *dfrA12*-*orfF*-*aadA2* in 24 species, and the type I carrying *dfrA1*-*orfC* in 16 species. More detailed information on distribution of the integrons among different species is shown in Table S2. The same type IV integron carrying *dfrA27* and *aac(6*’)*-Ib-cr* gene encoding resistance to trimethoprim and ciprofloxacin, respectively, has not been previously described in other bacteria. Detailed analysis of type IV integron indicates that: 1) the 3’-end and the corresponding attC site sequences of *aac(6*’)*-Ib-cr* were missing; 2) the attI site was appeared in the 5’ flanking region of *aac(6*’)*-Ib-cr* and in the middle of this integron sequence; and 3) an arr-3 cassette was probably deleted and left its attC site still before the *dfrA27* gene. Compared with class I integron in *E. coli* strain DJ33-7 (JF806489), the type IV integron here is probably formed by a recombination event (Figure 2).

### Association between drug resistance, class 1 integron presence and MLST genotype in *K. pneumoniae*

The 176 *K. pneumoniae* isolates consisted of 61.4% (108/176) multidrug-resistant strains, 21.6% (38/176) extensively drug-resistant strains and 1.7% (3/176) pandrug-resistant strains. Eighty of the 176 isolates (45.5%) were ESBL positive, and 57 isolates among the 90 isolates containing class 1 integrons were ESBL positive. The detailed information on the rates of drug resistance among the tested 176 *K. pneumoniae* isolates with and without class 1 integrons is shown in Table 2. Isolates positive for class 1 integrons were resistant to an average of 10.7 antibiotics, compared with 5.9 antibiotics for class 1 integron negative isolates. ESBL production and resistance to the following drugs was significantly higher among class 1 integron positive isolates than class 1 integron negative isolates: cefazolin, ceftriaxone, ceftazidime, cefepime, gentamicin, tobramycin, amikacin, streptomycin, spectinomycin, ciprofloxacin, levofloxacin, trimethoprim, piperacillin/tazobactam, ampicillin/sulbactam, and aztreonam. In contrast, resistance to cefotetan was significantly higher among class 1 integron negative isolates. The proportion of XDR isolates was significantly higher among class 1 integron positive isolates while the proportion of other types of isolates (non-MDR, non-XDR, and non-PDR isolates) were significantly higher among class 1 integron negative isolates. Furthermore, we found that the ESBL producing isolates with class 1 integrons had higher proportion of MDR (35.6% vs. 14.0%) and XDR (26.7% vs. 10.5%) isolates than those class 1 integron negative isolates.

**Table 2:** Rates of drug resistance among 176 *K. pneumoniae* isolates with and without class 1 integrons.

**Drugs**		**Class 1 integron positive isolates (n=90)**		**Class 1 integron negative isolates (n=86)**		***P* value**
		**Susceptible,n(%)**	**Resistant,n(%)**	**Susceptible,n(%)**	**Resistant,n(%)**	
Antimicrobial categories	With resistance to single drugs					
Cephalosporins	Cefazolin	28/90 (31.1)	62/90 (68.9)	60/86 (69.8)	26/86 (30.2)	<0.001
	Ceftriaxone	29/90 (32.2)	61/90 (67.8)	61/86 (70.9)	25/86 (29.1)	<0.001
	Ceftazidime	30/90 (33.3)	60/90 (66.7)	59/86 (68.6)	27/86 (31.4)	<0.001
	Cefepime	29/90 (32.2)	61/90 (67.8)	61/86 (70.9)	25/86 (29.1)	<0.001
Aminoglycosides	Gentamicin	34/90 (37.8)	56/90 (62.2)	66/86 (76.7)	20/86 (23.3)	<0.001
	Tobramycin	26/90 (28.9)	64/90 (71.1)	63/86 (73.3)	23/86 (26.7)	<0.001
	Amikacin	59/90 (65.6)	31/90 (34.4)	74/86 (86.0)	12/86 (14.0)	0.002
	Streptomycin	61/90 (67.8)	29/90 (32.2)	86/86 (100.0)	0	<0.001
Aminocyclitol antibiotic	Spectinomycin	64/90 (71.1)	26/90 (28.9)	86/86 (100.0)	0	<0.001
Carbapenems	Imipenem	83/90 (92.2)	7/90 (0.8)	84/86 (97.7)	2/86 (2.3)	0.101
	Ertapenem	82/90 (91.1)	8/90 (0.9)	81/86 (94.2)	5/86 (5.8)	0.436
Fluoroquinolones	Ciprofloxacin	39/90 (43.3)	51/90 (56.7)	66/86 (76.7)	20/86 (23.3)	<0.001
	Levofloxacin	40/90 (44.4)	50/90 (55.6)	67/86 (77.9)	19/86 (22.1)	<0.001
Folate pathway inhibitors	Trimethoprim	21/90 (23.3)	69/90 (76.7)	60/86 (69.8)	26/86 (30.2)	<0.001
Cephamycins	Cefotetan	34/90 (37.8)	56/90 (62.2)	20/86 (23.3)	66/86 (76.7)	0.037
Nitrofuran antibiotcs	Nitrofurantoin	9/90 (10.0)	81/90 (90.0)	15/86 (17.4)	71/86 (82.6)	0.150
Penicillins	Ampicillin	0	90/90 (100.0)	0	86/86 (100.0)	ND ^a^
Antipseudomonal penicillins+beta-lactamase inhibitors	Piperacillin/Tazobactam	57/90 (63.3)	33/90 (36.7)	78/86 (90.7)	8/86 (9.3)	<0.001
Penicillins+beta-lactamase inhibitors	Ampicillin/Sulbactam	30/90 (33.3)	60/90 (66.7)	60/86 (69.8)	26/86 (30.2)	<0.001
Ansamycins	Rifampicin	87/90 (96.7)	3/90 (3.3)	86/86 (100.0)	0	0.088
Monobactams	Aztreonam	28/90 (31.1)	62/90 (68.9)	62/86 (72.1)	24/86 (27.9)	<0.001
	Drug resistance types					
	MDR	36/90 (40.0)	54/90 (60.0)	32/86 (37.2)	54/86 (62.8)	0.704
	XDR	62/90 (68.9)	28/90 (31.3)	76/86 (88.4)	10/86 (11.6)	0.002
	PDR	89/90 (98.9)	1/90 (1.1)	84/86 (97.7)	2/86 (2.3)	0.534
	Other types	83/90 (92.2)	7/90 (7.8)	66/86 (76.7)	20/86 (23.3)	0.004
	ESBL production	33/90 (36.7)	57/90 (63.3)	63/86 (73.3)	23/86 (26.7)	<0.001

a ND: No data.

The detailed information for the distribution of sequence types and clonal complex among class 1 integron positive and negative isolates is shown in Table S3. The proportion of the isolates belonging to the following sequence types were higher among class 1 integron positive isolates than class 1 integron negative isolates: ST11 (7.8% vs. 3.5%), ST15 (14.4% vs. 7.0%), ST147 (11.1% vs. 0), ST562 (11.1% vs. 4.7%), and ST716 (11.1% vs. 2.3%). On the contrary, the proportion of the isolates belonging to ST23 was higher among class 1 integron negative isolates than class 1 integron positive isolates (12.8% vs. 3.3%).

## Discussion

The variation of the gene cassettes in class 1 integrons may reflect the horizontal transfer of integrons among members of the *Enterobacteriacea*e family. In this study, 10 different types of class 1 integron gene cassette arrays containing 14 gene cassettes were identified. The phenotypic resistance to a specific drug was observed in all isolates carrying the corresponding gene cassette. An integron of about 1,171bp with the *dfrA1* and *orfC* genes (the type I), which was present in 24 *K. pneumoniae* isolates, was the predominant integron gene cassette array detected. Except for type IV (*dfrA27-aac(6*’)*-Ib-cr*), all the other types of the class 1 integrons have been previously described in *E. coli* as well as in other members of the *Enterobacteriaceae* family [9,10,41-43]. To the best of our knowledge, this is the first report of detection of the gene cassette array *dfrA27-aac(6*’)*-Ib-cr* in class 1 integron from clinical *Enterobacteriaceae* isolates worldwide, and based on the sequence analysis results, we predict that this integron type might be formed by a complex recombinant event. Though the exact mechanism is unclear, we give our suggestions that part of sequence containing gene cassettes *aac(6*’)*-Ib-cr*, *arr3*, and *dfrA27* was probably cut from integrons similar to *E. coli* strain DJ33-7 class I integron, and then circled and deleted a 590bp fragment containing 3' end of *aac(6*')*-Ib-cr* and 5' end of *arr3* and integrated into downstream of a integrase. Remarkably, 2 integrons with amplicons of 1171 bp (*dfrA1-orfC*) and 1851 bp (*dfrA12-orfF-aadA2*) were detected in a single isolate. This isolate was isolated from an ICU patient with coronary heart disease. The isolate was an ESBL positive MDR strain with resistance to11 drugs (including cefazolin, ceftriaxone, ceftazidime, cefepime, ciprofloxacin, levofloxacin, nitrofurantoin, ampicillin, ampicillin/sulbactam, aztreonam, and trimethoprim), thus the spread of such strains is hazardous and should be controlled. 

In addition to previous reported gene cassettes (most of which are associated with drug resistance), we also identified 2 previously unreported potential gene cassettes (in class 1 integron type X, see Table 1 and Figure 1) including a gene encoding a hypothetical protein (with 41% identity with an uncharacterized amino acid transporter from an uncultured bacterium) and the *mfs-1* among class 1 integron from a single isolate. *mfs-1* encoding for a protein belong to the Major facilitator superfamily, which has been found to be associated with multidrug resistance among bacteria [44,45], but since the *mfs-1* we detected in the class 1 integron was partial, thus it is probably non-functional in this context. This type X integron has only been found in *K. pneumoniae* strain NF811347 (HQ880282). We further search the clinical information for this isolate and found that it was obtained from a one-month-old newborn baby suffered from pneumonia, and this isolate was resistant only to ampicillin, further suggesting that those two gene cassettes in the class 1 integron from this isolate are not associated with drug resistance. In addition, as no known obvious attC sites of these two gene cassettes were found in this integron, the incorporation mechanism of these cassettes in unclear. The observation of identical integrons with prevalent arrays in the genomes among diverse bacteria species of phylogenetically distant bacteria strongly suggests the occurrence of intergeneric horizontal transfer of genetic cassettes. However, issues concerning horizontal transfer and dissemination of integrons between Gram-positive and Gram-negative organisms, as well as the origins of class 1 integron still remained unclear and required further investigation.

It is well established that class I integrons play a major role in the dissemination of drug resistance in clinical bacterial isolates and the use of broad-spectrum antimicrobial agents has had a profound effect on promoting this process. We observed that those class 1 integron positive isolates exhibited resistance to a much higher number of drugs as compared with those class 1 integron negative isolates. The proportion of XDR isolates was significantly higher among class 1 integron positive isolates. We also found that the proportion of a variety of drug resistance genes, including those located within and outside of class 1 integrons, was significantly higher among class 1 integron positive isolates as compared to class 1 integron negative isolates. Some genes such as *aac(6*')-Ib-cr and aacA4 are often described within integron gene cassettes [46,47], it is interesting to find those genes outside of class 1 integrons in our study. Since some class 1 integrons do not contain the 3'-CS, we used the 5’ CS primer coupled to a primer specific for the aac(6')-Ib-cr gene or aacA4 gene for PCR amplification in order to confirm whether aac(6')-Ib-cr and aacA4 were located within class 1 integrons lacking the 3´ CS. Indeed, we found that 7 of the 9 aac(6')-Ib-cr containing isolates and 4 of the 13 aacA4 containing isolates whose class 1 integrons were not successfully detected by 3'CS and 5'CS primers had their aac(6')-Ib-cr genes or aacA4 genes in the partial class 1 integrons lacking the 3’ CS. Nevertheless, there were still isolates having their aac(6')-Ib-cr genes (n=7) or aacA4 genes (n=4) outside of the class 1 integrons, and these results are consistent with a couple of other studies in which aac(6')-Ib-cr genes were also identified outside of the class 1 integrons [48,49]. Those findings provided further evidence of the strong association between the presence of class 1 integrons and the acquisition of drug resistance and the corresponding genes, both within and outside of the class 1 integrons, among clinical K. pneumoniae Isolates. Another explanation for this association could be co-selection processes due to spread of successful clones or clonal lineages (such as ST11 and ST258 K. pneumoniae) that were selected by antibiotic treatment in the hospital settings and that were able to accumulate various resistance genes [50,51]. In this study, we further showed that the proportion of some previously identified prevalent sequence types were higher among class 1 integron positive isolates than class 1 integron negative isolates, such as ST11, ST15, ST147, ST562, and ST716, as described previously [13]. Since the prevalent clones have a great potential of transmission among patients, the observation that those prevalent clones are significantly associated with the presence of class 1 integrons suggest that those isolates could be a dangerous reservoir for transmission of drug resistance genes, thus warrant a high degree of awareness. We also noticed that carbapenemase genes were not detected in 6 of the 13 ertapenem resistant isolates, which suggest the presence of other mechanisms responsible for resistance against carbapenems in those *K. pneumoniae* isolates. For example, the combination of ESBL production and porin loss in those isolates could cause the development of carbapenem resistance [52,53].

In conclusion, data from this study suggest that class I integrons are highly diverse and are associated with a variety of drug resistance phenotypes, drug resistance genes, as well as clones and clonal lineages among *K. pneumoniae* isolates in hospital settings in Beijing, China. Accordingly, continued surveillance of the epidemiology as well as more in-depth investigation of characteristics of those integrons and their gene cassettes are warranted to provide updated information on the current situation and molecular mechanisms of acquisition of multiple drug resistance genes among *K. pneumoniae*, an important reservoir for the transmission of drug resistance among *Enterobacteriaceae.*

### Nucleotide sequence accession numbers

The nucleotide sequences of the ten types of integron gene cassette arrays reported in this study are deposited in the GenBank database under the accession numbers listed in Table 1 (JQ823008-JQ823017).

## Supporting Information

Table S1 **Percentage of drug resistance genes not located within class 1 integron gene cassettes in 176 *K. pneumoniae* isolates.**
(DOC)

Table S2 **Distribution of the detected class 1 integrons with prevalent arrays among diverse bacterial species.**
(DOC)

Table S3 **Distribution of sequence types and clonal complex among Class 1 integron positive and negative isolates.**
(DOC)
