# Supplementary material for: Structural Diversity of Class 1 Integrons and Their Associated Gene Cassettes in Klebsiella pneumoniae Isolates from a Hospital in China
Source: PLoS One. 2013 Sep 30;8(9):e75805. doi: 10.1371/journal.pone.0075805 (PMC3786929; doi:10.1371/journal.pone.0075805)
Supplement: Table S1 — Percentage of drug resistance genes not located within class 1 integron gene cassettes in 176 K. pneumoniae isolates. (DOC) [file pone.0075805.s001.doc]

| **Table S1.** Percentage of drug resistance genes not located within class 1 integron gene cassettes in 176 *K. pneumoniae* isolates. | | | | | | |
| --- | --- | --- | --- | --- | --- | --- |
|  | | | | | | |
| **Resistance gene categories** | **Resistance-associated genes** | **Class 1 integron positive isolates (n=90)** | | **Class 1 integron negative isolates (n=86)** | | ***P* value** |
|  |  | **Susceptible, n (%)** | **Resistant, n (%)** | **Susceptible, n (%)** | **Resistant, n (%)** |  |
| ESBL genes | *bla*_CTX-M-1_ | 69/90 (76.7) | 21/90 (23.3) | 79/86 (91.9) | 7/86 (8.1) | 0.006 |
|  | *bla*_CTX-M-2_ | 90/90 (100) | 0 | 84/86 (97.7) | 2/86 (2.3) | 0.146 |
|  | *bla*_CTX-M-3_ | 69/90 (76.7) | 21/90 (23.3) | 81/86 (94.2) | 5/86 (5.8) | 0.001 |
|  | *bla*_CTX-M-8_ | 76/90 (84.4) | 14/90 (15.6) | 83/86 (96.5) | 3/86 (3.5) | 0.007 |
|  | *bla*_CTX-M-9_ | 81/90 (90.0) | 9/90 (10.0) | 83/86 (96.5) | 3/86 (3.5) | 0.087 |
|  | *bla*_CTX-M-14_ | 73/90 (81.1) | 17/90 (18.9) | 77/86 (89.5) | 9/86 (10.5) | 0.115 |
|  | *bla*_CTX-M-15_ | 72/90 (80.0) | 18/90 (20.0) | 80/86 (93.0) | 6/86 (7.0) | 0.012 |
|  | *bla*_CTX-M-10_ | 52/90 (57.8) | 38/90 (42.2) | 65/86 (75.6) | 21/86 (24.4) | 0.012 |
|  | *bla*_CTX-M-25_ | 88/90 (97.8) | 2/90 (2.2) | 86/86 (100.0) | 0 | 0.164 |
|  | *bla*_CTX-M-55_ | 89/90 (98.9) | 1/90 (1.1) | 85/86 (98.8) | 1/86 (1.2) | 0.974 |
| AmpC bata-lactamase genes | *bla*_CMY-2_ | 88/90 (97.8) | 2/90 (2.2) | 82/86 (95.3) | 4/86 (4.7) | 0.375 |
|  | *bla*_DHA-1_ | 85/90 (94.4) | 5/90 (5.6) | 84/86 (97.7) | 2/86(2.3) | 0.273 |
|  | *bla*_FOX_ | 90/90 (100) | 0 | 86/86 (100) | 0 | ND ^a^ |
| Carbapenemase genes | *bla*_KPC-2_ | 88/90 (97.8) | 2/90 (2.2) | 86/86 (100.0) | 0 | 0.164 |
|  | *bla*_NDM-1_ | 90/90 (100.0) | 0 | 86/86 (100.0) | 0 | ND ^a^ |
|  | *bla*_IMP_ | 89/90 (98.9) | 1/90 (1.1) | 86/86 (100.0) | 0 | 0.327 |
|  | *bla*_VIM_ | 89/90 (98.9) | 1/90 (1.1) | 86/86 (100.0) | 0 | 0.327 |
|  | *bla*_OXA-48_ | 86/90 (95.6) | 4/90 (4.4) | 86/86 (100.0) | 0 | 0.048 |
| Further beta-lactamase genes | *bla*_TEM-1_ | 77/90 (85.6) | 13/90 (14.4) | 81/86 (94.2) | 5/86 (5.8) | 0.059 |
|  | *bla*_SHV-1_ | 66/90 (73.3) | 24/90 (26.7) | 70/86 (81.4) | 16/86 (18.6) | 0.202 |
|  | *bla*_SHV-11_ | 71/90 (78.9) | 19/90 (21.1) | 69/86 (80.2) | 17/86 (19.8) | 0.825 |
|  | *bla*_SHV-85_ | 86/90 (95.6) | 4/90 (4.4) | 78/86 (90.7) | 8/86 (9.3) | 0.201 |
|  | *bla*_TEM-186_ | 89/90 (98.9) | 1/90 (1.1) | 84/86 (97.7) | 2/86 (2.3) | 0.534 |
| PMQR genes | *qnrA* | 90/90 (100.0) | 0 | 84/86 (97.7) | 2/86 (2.3) | 0.146 |
|  | *qnrB* | 68/90 (75.6) | 22/90 (24.4) | 78/86 (90.7) | 8/86 (9.3) | 0.008 |
|  | *qnrC* | 89/90 (98.9) | 1/90 (1.1) | 86/86 (100.0) | 0 | 0.327 |
|  | *qnrD* | 66/90 (73.3) | 24/90 (26.7) | 80/86 (93.0) | 6/86 (7.0) | 0.001 |
|  | *qnrS* | 58/90 (64.4) | 32/90 (35.6) | 71/86 (82.6) | 15/86 (17.4) | 0.007 |
|  | *aac(6’)-Ib-cr* | 57/90 (63.3) | 33/90 (36.7) | 77/86 (89.5) | 9/86 (10.5) | <0.001 |
|  | *qepA* | 88/90 (97.8) | 2/90 (2.2) | 86/86 (100.0) | 0 | 0.164 |
| Aminoglycoside resistance genes | *aacA4* | 56/90 (62.2) | 34/90 (37.8) | 73/86 (84.9) | 13/86 (15.1) | 0.001 |
|  | *aacC1* | 87/90 (96.7) | 3/90 (3.3) | 85/86 (98.8) | 1/86 (1.2) | 0.334 |
|  | *aacC2* | 49/90 (54.4) | 41/90 (45.6) | 71/86 (82.6) | 15/86 (17.4) | <0.001 |
|  | *aadA1* | 62/90 (68.9) | 28/90 (31.1) | 75/86 (87.2) | 11/86 (12.8) | 0.003 |
|  | *aadB* | 87/90 (96.7) | 3/90 (3.3) | 85/86 (98.8) | 1/86 (1.2) | 0.334 |
|  | *aphA6* | 89/90 (98.9) | 1/90 (1.1) | 86/86 (100.0) | 0 | 0.327 |
|  | *armA* | 83/90 (92.2) | 7/90 (7.8) | 83/86 (96.5) | 3/86 (3.5) | 0.219 |
|  | *rmtB* | 87/90 (96.7) | 3/90 (3.3) | 85/86 (98.8) | 1/86 (1.2) | 0.334 |
| Trimethoprim resistance genes | *dhfr* | 57/90 (63.3) | 33/90 (36.7) | 74/86 (86.0) | 12/86 (14.0) | 0.001 |
|  | | | | | | |
