# Supplementary material for: Structural Diversity of Class 1 Integrons and Their Associated Gene Cassettes in Klebsiella pneumoniae Isolates from a Hospital in China
Source: PLoS One. 2013 Sep 30;8(9):e75805. doi: 10.1371/journal.pone.0075805 (PMC3786929; doi:10.1371/journal.pone.0075805)
Supplement: Table S2 — Distribution of the detected class 1 integrons with prevalent arrays among diverse bacterial species. (DOC) [file pone.0075805.s002.doc]

| **Table S2.** Distribution of the detected class 1 integrons with prevalent arrays among diverse bacterial species. | | | |
| --- | --- | --- | --- |
|  | | | |
| **Integron type (No. of isolates)** | **Gene cassette arrays** | **Accession No.** | **Similar integrons in other different bacteria**  **(GenBank accession No.)^a^** |
| I (24) | *dfrA1-orfC* | JQ823008 | *A. baumannii* str. AYE (CU459141), *A. caviae* isolate 9LO (FM207631), *A. encheleia* strain V46-T1 (JN645881), Bacterium AK-MB38 (AM997276), *C. freundii* (AY069972), *E. coli* (AB161449), *K. pneumoniae* strain NF761255 (JN108891), *L. hongkongensis* strain LHW280 (GU726909), *P. mirabilis* strain NF915770 (HQ880252), *P. aeruginosa* (DQ315789), *S. enterica* subsp. enterica serovar Emek (AY963803), *Salmonella* sp. H16-244 (AB186122), *S. marcescens* (DQ402098), *S. metamorpha* strain V40-T1 (JN645880), *V. cholerae* non-O1/non-O139 (AB219453), *V. fluvialis* strain BD73 (AY605688) |
| II (12) | *dfrA17-aadA5* | JQ823009 | Bacterium AK-MB25 (AM997274), *C. freundii* strain V43-G1 (JN645876), *E. cloacae* (EF571855), *E. coli* strain ZJ157 (EU687490), *K. pneumoniae* strain KF3 (FJ876827), *K. Georgiana* (FN568351), *M. morganii* strain V26-M1 (JN645879), *P. aeruginosa* (DQ838665), *S. enterica* subsp. (AB126604), *Salmonella* sp. S126 (AY263739), *S. flexneri* (FJ895301), *Shigella* sp. ER.1.23 (FJ460182), *S. epidermidis* (AB291061), *S. hominis* (AB291062), *S. maltophilia* strain 315 (GQ924479), Uncultured bacterium (AY139591) |
| III (10) | *dfrA12-orfF-aadA2* | JQ823010 | *A. baumannii* strain CO26-2005 (GU304661), *A. hydrophila* F54 (FM877483), *A. media* strain ER (FJ460175), *A. punctata* isolate 08019 (FM957886), Bacterium AK-MB40 (FM179328), *C. freundii* strain Cf817 (HM589046), *E. tarda* strain CK41 (HQ332785), *E. aerogenes* strain Q4079 (HQ401565), *E. cloacae* strain E79 (HM998987), *E. faecalis* (AB196348), *E. coli* strain DJ33-11 (JF806493), *K. pneumoniae* strain NF812951 (JN119853), *P. mirabilis* strain NF909281 (HQ880253), *P. rettgeri* (GU165831), *P. aeruginosa* (AB191047), *S. enterica* subsp. (HQ840942), *S. marcescens* (AF284063), *S. flexneri* (FJ895302), *S. aureus* (AB191048), *S. epidermidis* strain SEP8 (AB297447), *S. hominis* strian SHO6 (AB297448), *S. warneri* strain Swa1 (AB297450), *S. maltophilia* strian 10 (GQ981416), Uncultured bacterium clone WF16clone28 (GU060321) |
| IV (10) | *dfrA27-aac(6’)-Ib-cr ^a^* | JQ823011 | None |
| V (4) | *aadA2* | JQ823012 | *A. caviae* strain ER.1.9 (FJ460178), *A. hydrophila* plasmid pRA3 (DQ401103), *A. media* strain ER.1.11 (FJ460179), *A. salmonicida* plasmid pAr-32 (AJ517791), *Aeromonas* sp. MM.1.10 (EU089667), Bacterium NV 66 (FN396367), Bacterium OD 21 (FN396378), *C. jejuni* CIT134C (AF530636), *C. amalonaticus* isolate CA-1 (AF486817), *C. freundii* isolate CF-12 (AF458081), *C. glutamicum* strain ATCC31830 (AF164956), *E. aerogenes* plasmid pYMG-5 (FJ004895), *E. cloacae* (AB194701), *E. coli* (FJ855127), *K. pneumoniae* strain Kp1206 (EU622038), *L. hongkongensis* strain LHW339 (GU726913), *P. mirabilis* strain NF991579 (HQ880254), *P. aeruginosa* plasmid pSA1700 (D43625), *Pseudomonas* sp. Tik3 (FN821089), *R. anatipestifer* (AY968682), *S. enterica* subsp. isolate N80 (DQ133165), *S. enteritidis* plasmid pSAL-1 (AJ237702), *Salmonella* sp. S14 (AY263740), *S. typhimurium* DT104 (AF071555), *S. aureus* (AB253625), *S. epidermidis* (AB291063), Uncultured bacterium clone BF7C12HS298MRG292 (FJ820125), *V. cholerae* non-O1/non-O139 (AB219456), *Y. pestis* biovar Orientalis str. IP275 plasmid pIP1202 (CP000603) |
| VI (4) | *dfrA1-aadA1* | JQ823013 | *A. baumannii* (DQ112355), *A. allosaccharophila* strain ER.1.6 (FJ460177), *A. sobria* isolate F101-2 (FM877479), *A.* sp. AK-MB21 (AM937243), Bacterium AK-MB26 (AM991328), *E. aerogenes* (EF093145), *E. coli* strain DJ33-19 (JF806496), *K. pneumoniae* (AY007807), *P. mirabilis* (DQ221761), *P. fluorescens* strain d124 (FJ950724), *R. anatipestifer* (FJ711657), *S. enterica* subsp. enterica serovar Typhimurium (FN432031), *S. infantis* (AJ879461), *S. typhimurium* 8/03 phagetype U302 (EF204551), Uncultured bacterium plasmid pTc1 (AY115476) |
| VII (3) | *aac(6’)-Ib-cr-aar-3* | JQ823014 | *E. coli* strain DJ33-7 (JF806489), *K. pneumoniae* strain C2367 (JF775514), *S. rubidaea* plasmid pLC108 (GU165830) |
| VIII (2) | *dfrA25* | JQ823015 | *C. freundii* (AB280920), *K. pneumoniae* strian NF709910 (HQ880262), *S. enterica* subsp. enterica serovar Senftenberg (FN252408), *S. marcescens* strain CH1 (GU991999) |
| IX (1) | *aadA1* | JQ823016 | *A. xylosoxidans* strain K-407 (HQ832471), *A. media* strain ER.1.5 (FJ460176), *A. salmonicida* subsp. salmonicida A449 (CP000645), Bacterium AK-MB24 (AM991326), *D. indicum* S5 (CP002432), *E. cloacae* strain S-98 (GQ924774), *E. faecalis* plasmid pNCC801 (AF052459), *E. coli* plasmid p838C-R1 (HQ201416), *K. pneumoniae* strain Kp2400 (EU622040), *P. mirabilis* (AY677093), *S. enterica* subsp. enterica serovar Typhimurium (AB605179), *S. metamorpha* strain 21S-G4 (JN645874), *S. maltophilia* strain K-28 (HQ832470), Uncultured bacterium plasmid pRSB201 (JN102341), *V. cholerae* isolate CIRPS1007 (EU697605) |
| X (1) | ORF for hypothetical protein, *mfs-1 ^a^* | JQ823017 | *K. pneumoniae strain NF811347 class I integron (HQ880282)* |
| ^a^ Only one representative species is listed (query coverage>90%, NT identity >99%); ^b^ partial gene. | | | |
