# Supplementary material for: Structural Diversity of Class 1 Integrons and Their Associated Gene Cassettes in Klebsiella pneumoniae Isolates from a Hospital in China
Source: PLoS One. 2013 Sep 30;8(9):e75805. doi: 10.1371/journal.pone.0075805 (PMC3786929; doi:10.1371/journal.pone.0075805)
Supplement: Table S3 — Distribution of sequence types and clonal complex among Class 1 integron positive and negative isolates. (DOC) [file pone.0075805.s003.doc]

| **Table S3.** Distribution of sequence types and clonal complex among Class 1 integron positive and negative isolates. | | | |
| --- | --- | --- | --- |
|  | | | |
| **Clonal complex** | **Sequence type** | **Class 1 integron positive isolates (n=90)** | **Class 1 integron negative isolates (n=86)** |
| CC23 | 23 | 3 | 11 |
|  | 11 | 7 | 3 |
|  | 15 | 13 | 6 |
|  | 17 | 3 | 3 |
|  | 65 | 0 | 3 |
|  | 340 | 0 | 2 |
|  | 895 | 0 | 2 |
|  | 815 | 0 | 1 |
|  | 35 | 1 | 0 |
|  | 268 | 0 | 1 |
|  | 875 | 0 | 1 |
|  | 25 | 1 | 1 |
|  | 36 | 0 | 3 |
|  | 218 | 1 | 1 |
|  | 375 | 0 | 3 |
|  | 412 | 2 | 5 |
|  | 660 | 0 | 1 |
|  | 685 | 1 | 0 |
|  | 874 | 0 | 1 |
|  | 887 | 0 | 1 |
|  | 692 | 0 | 1 |
|  | 879 | 0 | 1 |
|  | 261 | 0 | 1 |
|  | 462 | 3 | 0 |
|  | 881 | 0 | 1 |
| CC37 | 37 | 4 | 2 |
|  | 896 | 0 | 3 |
|  | 884 | 2 | 0 |
|  | 880 | 1 | 0 |
| CC29 | 29 | 0 | 1 |
| CC101 | 101 | 0 | 1 |
|  | 888 | 1 | 0 |
| CC86 | 86 | 0 | 3 |
| CC1 | 1 | 0 | 1 |
| CC12 | 876 | 0 | 1 |
| CC147 | 147 | 10 | 0 |
|  | 273 | 2 | 2 |
| CC857 | 857 | 1 | 0 |
| CC584 | 584 | 1 | 0 |
| CC526-928 | 526 | 2 | 0 |
| CC536-856 | 856 | 0 | 1 |
| CC889-7 | 889 | 1 | 0 |
| Singleton | 894 | 0 | 1 |
| Singleton | 893 | 0 | 1 |
| Singleton | 892 | 1 | 0 |
| Singleton | 891 | 0 | 1 |
| Singleton | 890 | 0 | 1 |
| Singleton | 886 | 0 | 1 |
| Singleton | 883 | 1 | 0 |
| Singleton | 882 | 1 | 0 |
| Singleton | 878 | 0 | 2 |
| Singleton | 877 | 1 | 0 |
| Singleton | 189 | 1 | 0 |
| Singleton | 374 | 0 | 1 |
| Singleton | 380 | 1 | 2 |
| Singleton | 562 | 10 | 4 |
| Singleton | 629 | 1 | 0 |
| Singleton | 686 | 3 | 1 |
| Singleton | 716 | 10 | 2 |
| Singleton | 873 | 0 | 1 |
|  | | | |
